# Upstream perturbation and floodplain formation effects on chute‐cutoff‐dominated meandering river pattern and dynamics

**DOI:** 10.1002/esp.4638

**Published:** 2019-04-23

**Authors:** Steven A. H. Weisscher, Yasuyuki Shimizu, Maarten G. Kleinhans

**Affiliations:** ^1^ Faculty of Geosciences Utrecht University Princetonlaan 8A Utrecht 3584 CB The Netherlands; ^2^ Faculty of Engineering Hokkaido University North 13, West 8, Kitaku, Sapporo Hokkaido 080‐8628 Japan

**Keywords:** fluvial morphodynamics, meander migration, floodplain formation, perturbation, convective instabilities

## Abstract

A sustained dynamic inflow perturbation and bar–floodplain conversion are considered crucial to dynamic meandering. Past experiments, one‐dimensional modelling and linear theory have demonstrated that the initiation and persistence of dynamic meandering require a periodic transverse motion of the inflow. However, it remains unknown whether the period of the inflow perturbation affects self‐formed meander dynamics. Here, we numerically study the effect of the inflow perturbation period on the development and meander dynamics of a chute‐cutoff‐dominated river, which requires two‐dimensional modelling with vegetation forming floodplain on bars. We extended the morphodynamic model Nays2D with growth and mortality rules of vegetation to allow for meandering. We tested the effect of a transversely migrating inflow boundary by varying the perturbation period between runs over an order of magnitude around typical modelled meander periods. Following the cutoff cascade after initial meander formation from a straight channel, all runs with sufficient vegetation show series of growing meanders terminated by chute cutoffs. This generates an intricate channel belt topography with point bar complexes truncated by chutes, oxbow lakes, and scroll‐bar‐related vegetation age patterns. The sinuosity, braiding index and meander period, which emerge from the inherent biomorphological feedback loops, are unrelated to the inflow perturbation period, although the spin‐up to dynamic equilibrium takes a longer time and distance for weak and absent inflow perturbations. This explains why, in previous experimental studies, dynamic meandering was only accomplished with a sustained upstream perturbation in flumes that were short relative to the meander wavelength. Our modelling of self‐formed meander patterns is evidence that scroll‐bar‐dominated and chute‐cutoff‐dominated meanders develop from downstream convecting instabilities. This insight extends to many more fluvial, estuarine and coastal systems in morphological models and experiments, which require sustained dynamic perturbations to form complex patterns and develop natural dynamics. © 2019 The Authors. Earth Surface Processes and Landforms Published by John Wiley & Sons Ltd.

## Introduction

**Figure 1:**
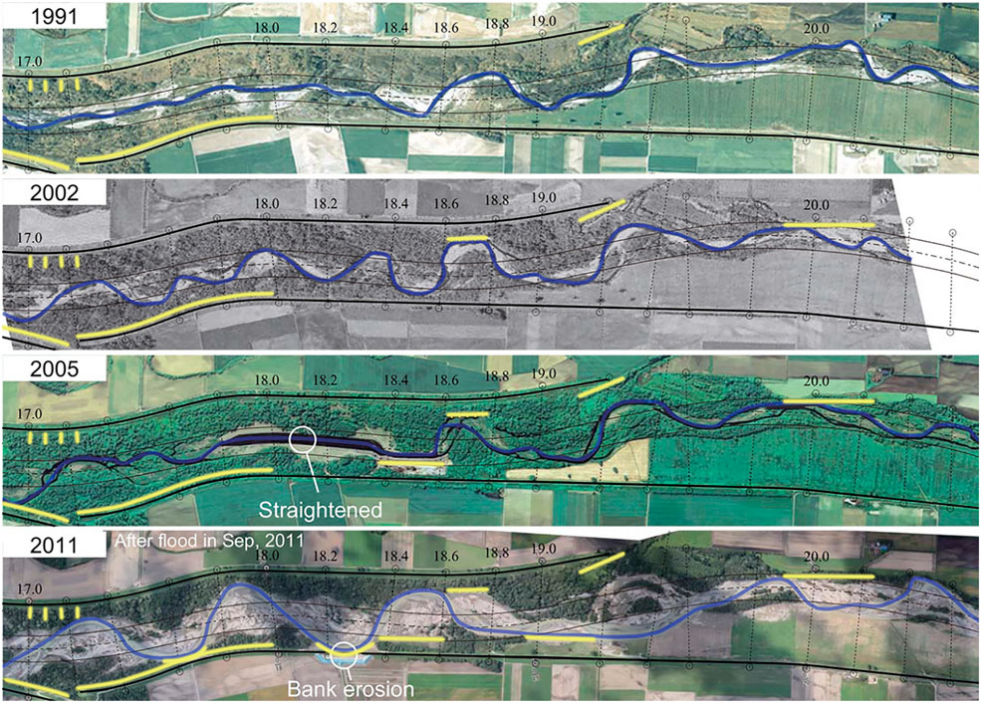
Time series of the River Otofuke in Hokkaido, Japan. Dikes (thick black lines) border the active channel belt and the thin black lines show the course of the river in the 1970s, after having been artificially straightened and widened. Scattered dikes and groynes constructed in the floodplain are given in yellow. Numbers indicate the distance in kilometres from a downstream confluence. Flow is from the right to the left. (Modified from Nagata et al., 2013.) [Colour figure can be viewed at wileyonlinelibrary.com]

A meandering river forms an elaborate floodplain topography through meander initiation, expansion and cutoff (Hickin and Nanson, 1975; Hooke, 2004). The rate at which meanders migrate is commonly described as a function of bend sharpness, in which the migration rate increases towards an optimum and subsequently drops with increasing bend sharpness (e.g. Hickin and Nanson, 1975; Furbish, 1988; Crosato, 2009). While this approach focuses on individual bends, it is evident, for example from the River Otofuke in Japan (Figure 1) and the River Allier in France (Kleinhans and Van den Berg, 2011), that the migration rate of one bend affects that of its downstream neighbours. Current understanding of such spatiotemporally radiating effects is that bend instabilities propagate in one – usually the downstream – direction rather than in both directions. However, the question is whether the period of such lateral instabilities, relative to the meander period, affects the degree of meandering dynamics. Furthermore, the implication of the downstream propagation of bend instabilities is that perturbations need to be imposed on the upstream boundary of all numerical models and experiments to maintain dynamic meandering (Lanzoni and Seminara, 2006; Van Dijk *et al.* 2012; Weiss, 2016). Yet, it remains unclear what the period of such perturbations would need to be and whether that affects the modelled meander migration rate and chute cutoff frequency.

Floodplain formation is thought to be the main necessary condition for dynamic meandering. However, it is the intricacies of floodplain formation that makes meandering notoriously challenging to be reproduced by numerical models (Nicholas, 2013; Van Oorschot *et al.* 2016; Schuurman *et al.* 2016) and laboratory landscape experiments (Braudrick *et al.* 2009; Tal and Paola, 2010; Van Dijk *et al.* 2012; Van Dijk *et al.* 2013). In the context of this study, a landscape experiment is a simulation of a fluvial, estuarine or coastal landscape with sediment mobility, morphodynamic processes and complexity comparable to those of natural systems (Kleinhans *et al.* 2014). A landscape experiment is considered intermediate between a classic scale model by similarity rules and an analogue model. In the absence of bank protection, unhindered widening results in bend cutoffs, mid‐channel bars and braiding (Parker, 1976). In contrast, to maintain a single‐thread channel that can form meanders, the width‐to‐depth ratio of the channel needs to be small enough to inhibit the onset of mid‐channel bars (Parker, 1976; Struiksma *et al.* 1985; Crosato and Mosselman, 2009; Kleinhans and Van den Berg, 2011), which requires that outer bend erosion is balanced by inner bend accretion (Van de Lageweg *et al.* 2014). Meandering may result from some form of strength in eroding outer banks (e.g. Leopold and Wolman, 1957; Ferguson, 1987; Kleinhans and Van den Berg, 2011), but recent modelling and past experiments suggest that meandering mainly requires floodplain formation on the point bar (see for review Solari *et al.*, 2016). Accordingly, this encroachment on the point bar causes the focus of flow into the outer bend and reduces the tendency to incise chutes on the point bar that is the onset of braiding (Braudrick *et al.* 2009; Van Dijk *et al.* 2012; Van Oorschot *et al.* 2016; Kleinhans *et al.* 2018). Although floodplain formation occurs through floodplain sedimentation, vegetation settlement and the combination thereof, this study focuses on riparian vegetation as the bar–floodplain conversion agent.

Riparian vegetation interacts with fluvial morphodynamics in several ways. Firstly, added hydraulic drag by vegetation on the bars and floodplain causes flow to focus in a single‐thread channel (Van Oorschot *et al.* 2016). Secondly, vegetation enhances sediment deposition (Zong and Nepf, 2011; Corenblit *et al.* 2016; Kleinhans *et al.* 2018), thereby contributing to the vertical and lateral accretion of point bars (Stella *et al.* 2011; Van Dijk *et al.* 2013). Thirdly, riparian vegetation increases bank stability via roots and the production of soil organic matter, which reduce outer bend erosion (Simon and Collinson, 2002; Braudrick *et al.* 2009; Tal and Paola, 2010). Local conditions determine the settling, growth and mortality of vegetation, while the feedback of vegetation on the hydromorphological pattern modifies these conditions and the resulting spatial and age distribution of vegetation (Simon and Collinson, 2002; Van Oorschot *et al.* 2016). Most two‐dimensional morphodynamic model studies that incorporated vegetation parametrized it as static (see for review Solari *et al.*, 2016); there are only two studies that applied dynamic vegetation, allowing multiple tree taxa to settle, grow and perish based on local hydraulic conditions (Van Oorschot *et al.* 2016; Kleinhans *et al.* 2018). This advancement resulted in a chute‐cutoff‐dominated meandering pattern with a spatial and age distribution of vegetation similar to that in nature. However, these models initiated with a meandering topography and had a relative short domain relative to the meander wavelength, because the necessary conditions for sustained meandering were not studied.

The second necessary condition for sustained dynamic meandering in a model or experimental domain of limited length is a sustained dynamic upstream perturbation. Perturbations migrate and decay in the downstream direction as convective instabilities, as opposed to bidirectional absolute instabilities (Lanzoni and Seminara, 2006). Consequently, the dynamics of meandering rivers are found to decrease when no new perturbation is introduced at the upstream boundary, as observed in previous studies (e.g. Friedkin, 1945; Braudrick *et al.* 2009; Asahi *et al.* 2013). Conversely, landscape experiments showed that applying a periodic transverse inflow perturbation leads to dynamic chute‐cutoff‐dominated meandering. This novel concept was adopted in few two‐ dimensional models (Nicholas, 2013; Schuurman *et al.* 2016; Van de Lageweg *et al.* 2016), which enabled the development of high‐amplitude dynamic meandering with neck cutoffs. Yet, some of these models oversimplify bar–floodplain conversion and exclude the self‐formed floodplain topography from the flow by a minimum depth criterion, rendering these models quasi one‐dimensional. However, spatial differences on the vegetated bars and floodplain are conducive to the development of chute cutoffs and determine the location thereof (e.g. Fisk, 1947; Gay *et al.* 1998). This is crucial because chute cutoffs considerably limit sinuosity, and the impossibility thereof in one‐dimensional and most two‐dimensional models means that such models tend more easily to meandering than two‐dimensional models with unconstrained fluvial plains that can be entirely reworked and reactivated. Moreover, the effect of the period of a periodic transverse inflow perturbation on the dynamics has not been tested in two‐dimensional models and experiments. While absence of upstream perturbations reduces the dynamics and a slow perturbation perhaps simply acts as a weak forcing, it could be argued that relatively fast upstream perturbations are merely generating noise that causes braiding in the downstream direction. This reasoning leads to the hypothesis that there is an optimum perturbation rate at which meandering is most dynamic.

The objective of this study is to determine the effect of a periodic inflow perturbation on the river pattern and dynamics. To this end, we modelled a dynamic chute‐cutoff‐dominated meandering river with a self‐forming vegetated floodplain using the morphodynamic model Nays2D. We initiated the two‐dimensional modelling with a straight channel on a rectangular fluvial plain with vegetation settling and with a domain length of about ten bends long, i.e. much larger than the typical meander wavelength. The inflow boundary was laterally moved at a range of celerities and the resulting meandering characteristics and dynamics were quantified.

The idealized model settings were inspired by the River Allier in France, located in a nature conservation area between Vichy and Moulins (Geerling *et al.* 2006; Kleinhans and Van den Berg, 2011), by the strongly managed River Otofuke in Japan (Nagata *et al.* 2014; Iwasaki *et al.* 2016), and by past landscape laboratory experiments by the Kleinhans research group (Table 1). Both these rivers and the experiment classify as meandering gravel‐bed rivers with chute cutoffs. The Allier is one of few intermediately sized rivers in Europe that presently meanders freely and has natural discharge and sediment input variations, with peak discharge in winter. In contrast, the discharge of the Otofuke is partly regulated by a dam in its main tributary and strongly influenced by typhonic discharges in late summer, which occur on a decadal scale. Additionally, the river is laterally restricted by dikes bordering the active channel belt. While the Otofuke is allowed to meander and does show chute cutoffs, measures are frequently taken when expanding meanders threaten bank stability (Figure 1). These measures included the construction of local dikes and groynes in the active floodplain. However, meanders quickly propagated in the downstream direction along such artificial obstacles and expanded significantly at locations where such obstacles were lacking, as has occurred during typhoon‐induced floods in 2011 and 2016. Furthermore, measures were frequently destroyed during floods, so that all measures collectively provide spatiotemporally dynamic perturbations in the form of local topographic forcing for the meandering channel. The effect of this remains unclear and future river management of repeated interventions could benefit from a better understanding of such convection of spatial perturbations.

**Table 1:** Comparison between the naturally meandering River Allier (FR), the managed River Otofuke (JP), the landscape experiment by Van Dijk et al. (2012) and the river modelled on a laboratory scale of this study

Parameter	Unit	Allier	Otofuke	Van Dijk *et al.* (2012)	This study
Mean annual flood discharge	m^3^ s^−1^	500	161	1×10^−3^	6.4×10^−3^
Grain size	m	5×10^−3^	60×10^−3^	0.51×10^−3^	0.76×10^−3^
Channel width	m	75	25	0.3	0.5
Channel depth	m	1.2	1.8	0.015	0.04
Valley gradient	m m^−1^	3.3×10^−3^	6.7×10^−3^	5.5×10^−3^	2×10^−3^
Aspect ratio (width/depth)	—	62.5	13.9	20	12.5
Shields mobility number	—	0.06	0.09	0.07	0.07
Froude number	—	0.18	0.67	0.58	0.4

## Morphodynamic Model Setup

### Model description

We modelled a dynamic meandering river on a laboratory scale using Nays2D, starting from a straight channel. Nays2D is a physics‐based numerical model that solves the depth‐averaged nonlinear shallow water equations and computes bedload transport and bed‐level change (see Shimizu *et al.*, 2013, for equations and numerical implementation), and it is predominantly used in fluvial research on both a natural and laboratory scale (e.g. Asahi *et al.* 2013; Van de Lageweg *et al.* 2016; Schuurman *et al.* 2016). Bedload transport was computed with the Meyer‐Peter and Müller (1948) predictor. Sediment input at the inflow boundary was at local transport capacity in our idealized model to mitigate unwanted effects of sediment deficit, which neglects possible effects in the River Otofuke due to upstream dams. The effect of secondary flow on the sediment transport direction was parametrized from horizontal flow curvature (Engelund, 1974) to account for the three‐dimensional effects of flow. Gravitational bank erosion processes were captured by a slope collapse module (Iwasaki *et al.* 2016) that administered a bed elevation correction in case of slope oversteepening while maintaining mass conservation. To generate a periodic inflow perturbation, the position of the grid cells with inflow was moved transversely at a constant displacement rate with a maximum amplitude of 0.75 m, similar to Van Dijk *et al.* (2012), while maintaining a constant inlet width and slope angle of its banks. The computational domain was 4.5 m wide and 30 m long, and the amplitude of the inflow perturbation was much smaller than the typical meander amplitude.

Vegetation colonization and mortality were added to Nays2D. Every spring, vegetation colonized dry cells (i.e. with a water depth *h*<3 mm) and was removed from the remaining cells when drowned. A second means of mortality was scour, which was tested year‐round; vegetation was cleared away when the erosion depth exceeded the rooting depth (Table 2). The latter predominantly occurred on the outer bank of expanding meander bends, where bank failure exposed the roots. However, usually only part of the roots was exposed by bank failure, and the plant drowned and was subsequently removed the next spring. In our model, vegetation did not influence the critical angle for bank failure, and therefore meander migration was not limited in scenarios with very dense vegetation. The hydraulic drag that vegetation exerted on water flow was computed as follows, considering vegetation non‐submerged under all flow conditions (Baptist *et al.* 2007):

(1) $$C=\frac{1}{\sqrt{\frac{1}{C_{b}^{2}}+\frac{C_{D}mDh}{2g}}}$$

where *C* is the Chézy roughness coefficient including vegetation (m^0.5^ s^−1^), *C*_*b*_ is the Chézy roughness of the bed (m^0.5^ s^−1^), *C*_*D*_ is the drag coefficient (–), *m* is the stem density (m^−2^), *D* is the stem diameter (m), *h* is the water depth (m) and *g* is the gravitational acceleration (9.81 m s^−2^). Since we were not interested in the dynamics caused by vegetation life stages (Van Oorschot *et al.* 2016), vegetation density, drag and rooting depth were taken to be constant, i.e. age independent (see Table 2).

**Table 2:** Default initial and boundary conditions

Parameter	Value	Unit
Discharge *Q* (low–high)	2–6.4	L s^−1^
Time step hydrodynamics/bed level change	0.02	s
Time step vegetation settling	50	min
Grain size	0.76×10^−3^	m
Valley slope	2×10^−3^	m m^−1^
Inflow migration period *T*	170	h
Inflow migration amplitude	0.75	m
Drag coefficient vegetation *C*_*D*_	1	—
Vegetation stem thickness *D*	0.5×10^−3^	m
Rooting depth	0.03	m
Manning's *n*	0.02	s m^1/6^

### Model scenarios

A fixed rectilinear computational grid was used with 0.1 m square grid cells. The initial straight channel was 0.5 m wide and 0.04 m deep. The inlet had an initial lateral offset of +0.4 m, a fixed width of 0.5 m and fixed bank slopes of 45°, and inlet displacement commenced in the direction away from the valley axis. As the inlet was shifted laterally, the width‐to‐depth ratio of the inlet channel varied slightly, causing a minor oscillation of sediment input. We prescribed 432 cycles of a 50 min hydrograph that was varied between 2 and 6.4 L s^−1^. Each cycle represents a year in which the flow peaks in late summer, similar to the River Otofuke. In winter and spring, flow was near the beginning of motion.

The scale issue of sediment mobility must be resolved when a river is scaled down to a laboratory scale. Generally, in physical scale experiments, a grain size is used that is larger than expected from the characteristic length scale of the channel to prevent cohesion and other scale effects, while the smaller water depth results in lower flow velocity and thus in lower sediment mobility (Kleinhans *et al.* 2014). This discrepancy is overcome by employing a steeper valley gradient (e.g. Braudrick *et al.* 2009; Tal and Paola, 2010; Van Dijk *et al.* 2012). In this study, both the river dimensions and grain size were scaled down by a factor 80, which resulted in a uniform grain size *d*_50_=0.76 mm that was used in previous numerical models of the River Otofuke on a laboratory scale (Iwasaki, pers. comm.). This scaling factor is consistent with the linear grain size dependence of the Shields mobility number, but the time scaling from 365 days to 50 min in our model is a factor of 10 500, which is over two orders larger. Timescales are problematic to determine, for they emerge from sediment transport and meander dynamics (Kleinhans *et al.* 2015). Furthermore, the settling of vegetation every year implies and imposes a rate of morphodynamics. Returning to the issue of sediment mobility in our case, a relatively steep valley gradient of 0.002 m m^−1^ was applied to attain a sediment mobility of similar magnitude to that in the rivers Otofuke and Allier.

We conducted two series of scenarios, and several additional runs to test the effect of grid resolution (Table 3). Firstly, vegetation density was varied between 0 and 10 stems cm^−2^ to determine its effect on the channel pattern and dynamics, with the objective of selecting a run with a sustained dynamic meandering river for the inflow perturbation tests. Most of these scenarios were run briefly for reasons of computational cost. Secondly, an array of inflow perturbation rates was applied to a model run with the selected vegetation density to examine whether the imposed perturbation rate forced the river dynamics. This included a control run without a dynamic inflow perturbation (N9). Additional scenarios were run to study the effect of a finer grid with 0.05 m square grid cells, while 0.1 m was the default resolution. To this end, we selected three scenarios, namely the default run (N1F), and the runs with largest vegetation density (N8F) and second fastest inflow perturbation (N13F), to test grid resolution dependence and to examine whether similar morphology and dynamics occur in extreme vegetation and perturbation cases. Data reduction as explained below was done in parallel with that of the coarse‐grid scenarios.

**Table 3:** Model scenarios. N1 is the default scenario. Three scenarios that were also run on a finer grid with 5×5 cm square grid cells are denoted by an ‘F’

Scenario	Stem density *n* (cm^−2^)	Inflow period *T* (h)
N1/N1F	2.5	170
*Vegetation density scenarios*		
N2	0	170
N3	0.1	170
N4	0.5	170
N5	1.28	170
N6	3.72	170
N7	5	170
N8/N8F	10	170
*Inflow perturbation rate*		
N9	2.5	Fixed
N10	2.5	200
N11	2.5	127.5
N12	2.5	85
N13/N13F	2.5	42.5
N14	2.5	4.25

### Data analysis

The channel pattern and dynamics were qualitatively characterized by map comparisons of the bathymetry and of the spatial and age distribution of the vegetation, and quantitatively compared for the braiding index, sinuosity and meander period. Bathymetries were first detrended with the initial valley gradient. The upstream and downstream 5 m (50 cells) were disregarded to exclude boundary effects in case domain length‐averaged parameters were computed. The braiding index was calculated as the spatially averaged number of channels that exceeded the mean cross‐sectional Shields stress of the initial straight channel during low flow in each cross‐section of the grid. Sinuosity was defined as the distance along the main channel relative to the domain length. The main channel was found as the filtered path of the minima of a map calculated as detrended bed elevation times flow velocity to the power three, which was empirically found to be the best indicator of channel position that excludes fast overbank flow and deep but abandoned channels. The meander period was determined on the lateral motion of seven cross‐sections along the valley, and it was calculated as twice the time interval between two consecutive valley axis crossings, while filtering out intervals shorter than 5 h and manually adding crossings in rare cases where the valley axis was not crossed by the migrating channel. The spin‐up of the models was disregarded for determining the median and temporal variation of sinuosity, braiding index and meander period under dynamic equilibrium conditions; more specifically, in the initial stage with pristine floodplain, bends are still quasi regular and cut through at approximately the same time. Following this sinuosity peak, the more irregular meanders cut through at different times to result in an overall lower sinuosity at dynamic equilibrium, as also found in Camporeale *et al.*(2005).

## Results

### General development

**Figure 2:**
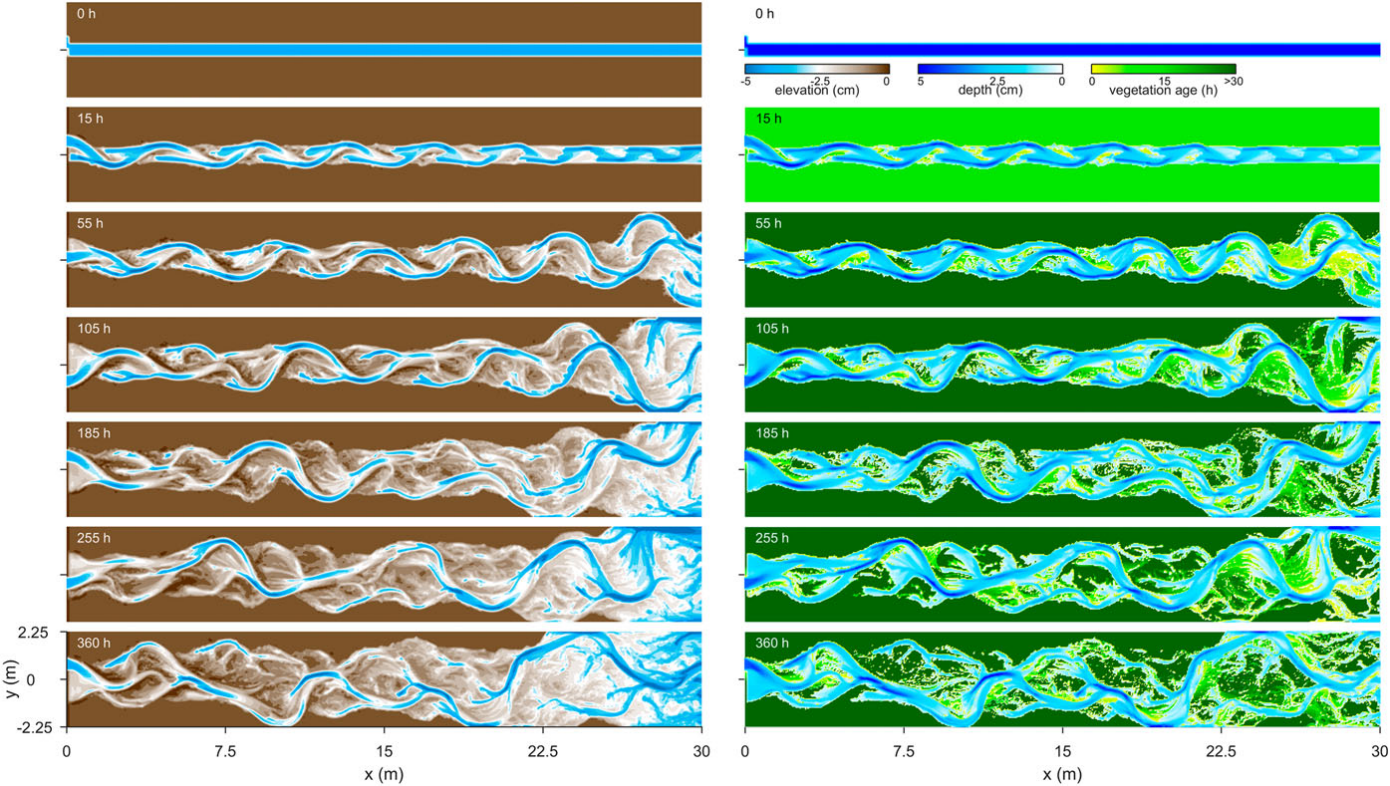
Development of river morphology in the default run N1F. Flow is from left to right. Left: bed elevation maps (DEMs) detrended by the initial valley slope. Right: water depth overlain by vegetation age [Colour figure can be viewed at wileyonlinelibrary.com]

The fine‐grid run of the default scenario (N1F), with intermediate stem density and inflow perturbation period, illustrates the development of the modelled dynamic meandering rivers with associated vegetation age patterns (Figure 2; Movie S1, supporting information). Initially, the development of a non‐migrating bar near the inlet caused localized erosion at the opposite bank, from which an alternate bar pattern advanced rapidly in the downstream direction. Subsequently, continued bank erosion, inner bank accretion and vegetation settling led to an increase in meander amplitude in the downstream direction that greatly exceeded the amplitude of the inflow perturbation. Meanwhile, the channel narrowed and deepened on the transition from alternate bars to meandering. Vegetation colonized the point bars as they expanded and migrated, which generated a scroll‐bar‐related vegetation age pattern. Clearly, a sustained meandering pattern formed from an initially straight channel in the applied conditions with floodplain and sustained upstream perturbation.

The chute cutoffs developed as follows. Since the mean wavelength of the meanders grew larger than that of the initial alternate bars, bends increasingly tightened. This bend sharpening reduced the channel gradient and caused increasing overbank flow. In consequence, incipient chutes formed at the upstream part of point bars, extended in the downstream direction and eventually cut off meander bends. The cutoff location on the point bar varied considerably, from chutes well across the entire point bar, far from the channel, to chutes through swales much nearer to the active channel. Interestingly, the river pattern changed intermittently between a weakly braided and a low‐amplitude sinuous channel, which depended on the number of cutoffs, while the boundary conditions remained constant. Despite the occurrence of multiple cutoffs, the river generally tended towards a single‐thread channel with up to 10 bends (Figure 2).

**Figure 3:**
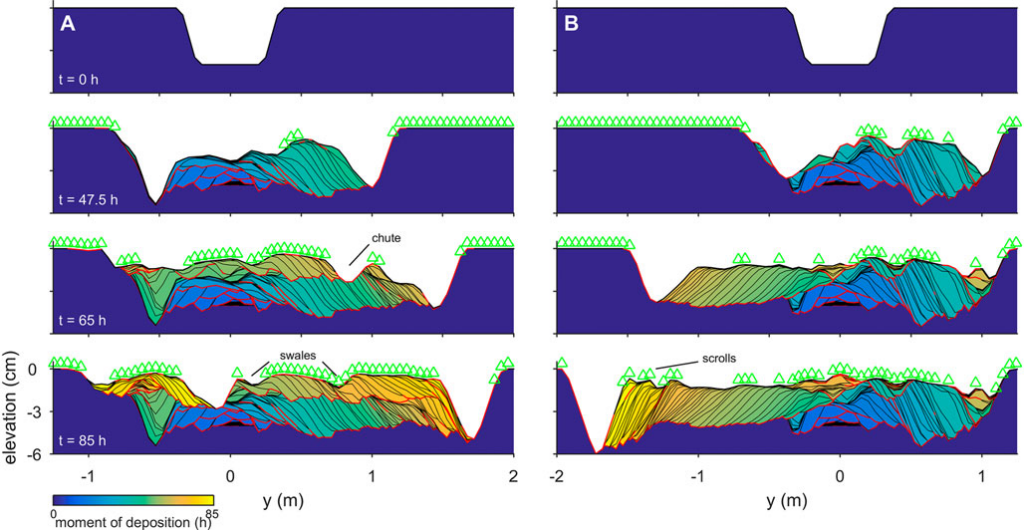
Examples of point bar development with lateral accretion surfaces, scrolls, swales and infill of a residual channel, which are indicative for meandering rivers. (a) Default model run N1F at x=23.65 m. (b) Model run N13F (fast upstream perturbation) at x=22.85 m. Vegetation is indicated by green triangles. Accretion surfaces and erosive contacts are indicated as black and red lines, respectively. The vertical exaggeration is 10 [Colour figure can be viewed at wileyonlinelibrary.com]

**Figure 4:**
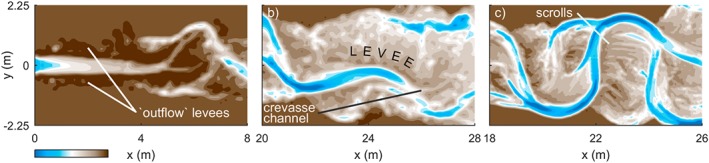
Illustrations of specific floodplain processes. (a) Levee formation along the channel just downstream of the fixed inflow location in scenario N9 at t=332.5 h. (b) Outer bend levee formation on top of former point bar complexes by repeated splay deposits in scenario N12 (T=85 h) at t=332.5 h. (c) Scroll bars on a point bar of a highly sinuous channel section in fine grid scenario N13F (T=42.5 h) at t=235 h. The bifurcation at x=24 m persists for a duration of ±45 h. Flow is from left to right [Colour figure can be viewed at wileyonlinelibrary.com]

The series of meander growth and cutoffs produced a complex morphology that was reflected in the stratigraphy of the channel belt (Figure 3). For example, the development of a point bar was preserved as a set of lateral accretion surfaces, which are interpreted as non‐erosive contacts in the sedimentological record and are considered characteristic of meandering river deposits. Abandoned meander bends were preserved as oxbow lakes in the channel belt and appeared to be filled in by splay deposits only when a chute or channel was in close proximity. When unfilled, such inactive channels sometimes reactivated by connecting with a chute at a later stage. In some model runs with a wide reworked floodplain, splay deposits contributed to the development of large outer bend levees (Figure 4b). Erosive contacts were formed by the initiation and migration of chutes and channels as they reworked sediment of older deposits. For instance, the chute channel at *y*=0.8 m in Figure 3a (*t*=65 h) eroded the top set of a still‐expanding point bar deposit and replaced it with a new point bar deposit before merging with the former main channel. Thus, single‐generation meander bends occasionally had more than one set of lateral accretion surfaces.

### Effect of vegetation density

**Figure 5:**
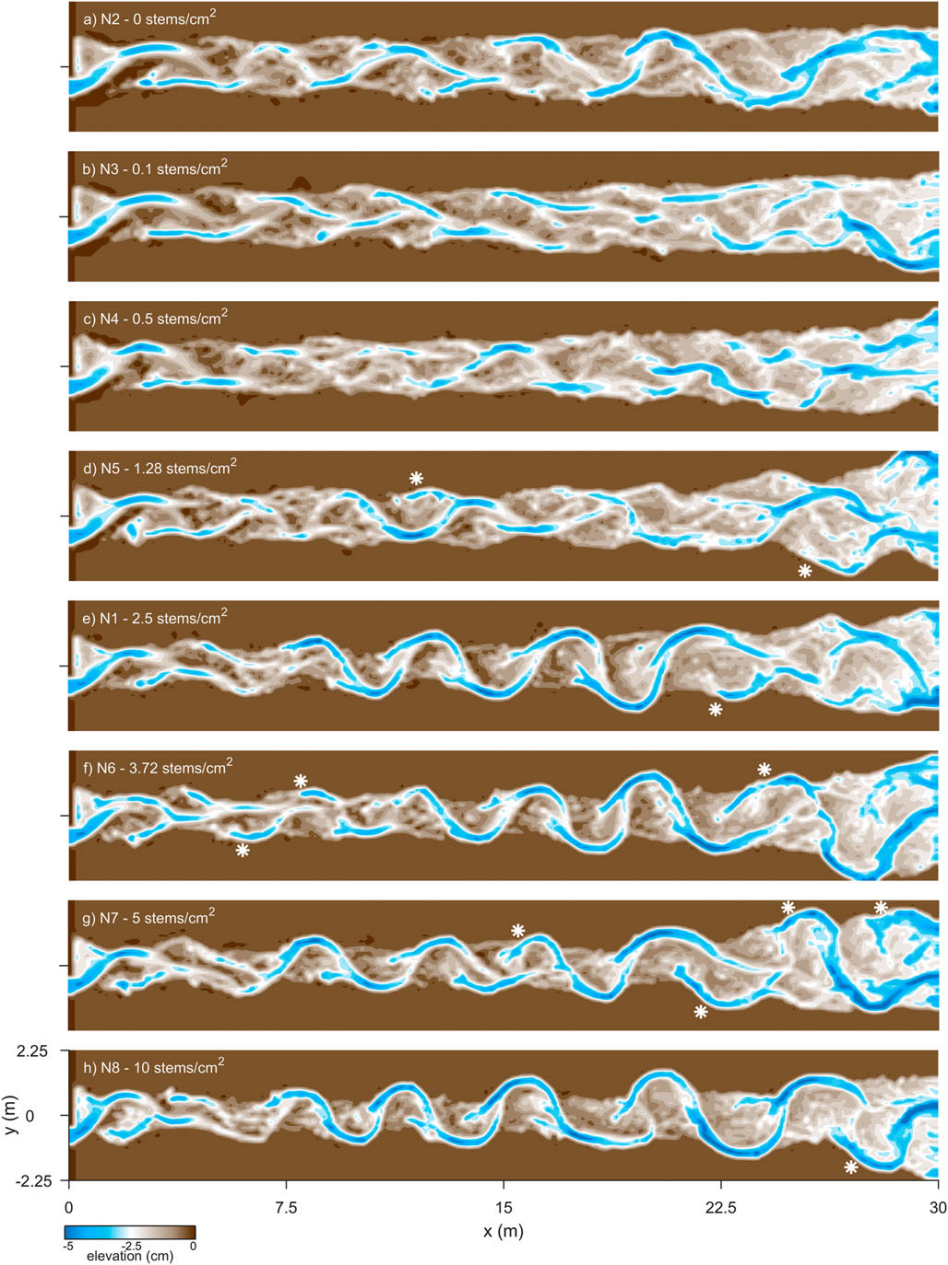
Maps of detrended bed elevation at t=100 h for model runs with increasing vegetation density from top to bottom, showing a transition from weakly braided to meandering with chutes. Infrequently formed meander bends in (a)–(d) are generally short‐lived. Examples of oxbow lakes are given as asterisks (*) in (d)–(h) [Colour figure can be viewed at wileyonlinelibrary.com]

**Figure 6:**
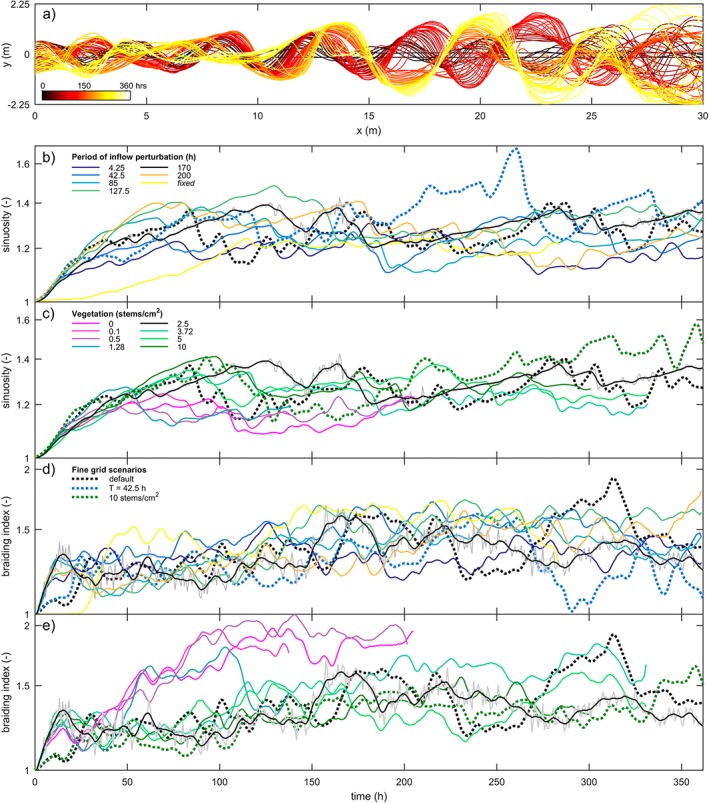
Quantification of river dynamics. (a) Channel centre lines of default run N1 showing meander extension, migration and chute cutoff. Time series of sinuosity for (b) different inflow perturbation periods and (c) vegetation density. Time series of braiding index for (d) different inflow perturbation periods and (e) vegetation density. Grey line for N1 illustrates sinuosity and braiding index before smoothing for clarity of presentation. Fine grid scenarios conducted to show resolution independence of results are shown as dashed lines (see legend in d) [Colour figure can be viewed at wileyonlinelibrary.com]

Increasingly dense vegetation reshaped the river pattern from shallow weakly braided to low‐amplitude meandering with chutes (Figure 5). Accordingly, the braiding index decreased from about 1.85 to 1.4, on average, with a rather sharp transition at 1.28 stems cm^−2^ (Figure 6). Temporary higher braiding indices correlated with the development of chutes and the reactivation of former channels preceding (failed) chute cutoff. Furthermore, the sinuosity increased with vegetation density and showed larger variability due to meander expansion and cutoff. Generally, sinuosity first overshot before reaching a dynamic equilibrium between 1.15 and 1.4. There was a slight overestimation of sinuosity for the weakly braided rivers, i.e. with a vegetation density ≤0.5 stems cm^−2^, because the extracted main channel was the path of the most dominant channel in a network with, on average, two channels in the cross‐section. In the most sinuous and densely vegetated channels, the meanders grew by localized erosion in the outer bends, which resulted in lateral expansion of the bends, followed by temporary semicircular scarps at the outer boundaries of the active channel belt (Figures 5e to 5h). Cutoff of these bends resulted in oxbow lakes that were predominantly located at the edges of the channel belt. In contrast, less sinuous channels with sparse vegetation eroded the outer banks more gradually, and the bends that formed were short‐lived and migrated in the downstream direction (Figures 5a to 5d). Generally, dense vegetation hampered the onset of braiding by quickly colonizing plug bars and by redirecting flow into the main channel.

To conclude, dynamic and sustained meandering rivers formed in scenarios with a vegetation density ≥2.5 stems cm^−2^. Of these scenarios, scenario N1 (2.5 stems cm^−2^) was used to test the effect of the inflow perturbation period.

### Effect of the inflow perturbation period

**Figure 7:**
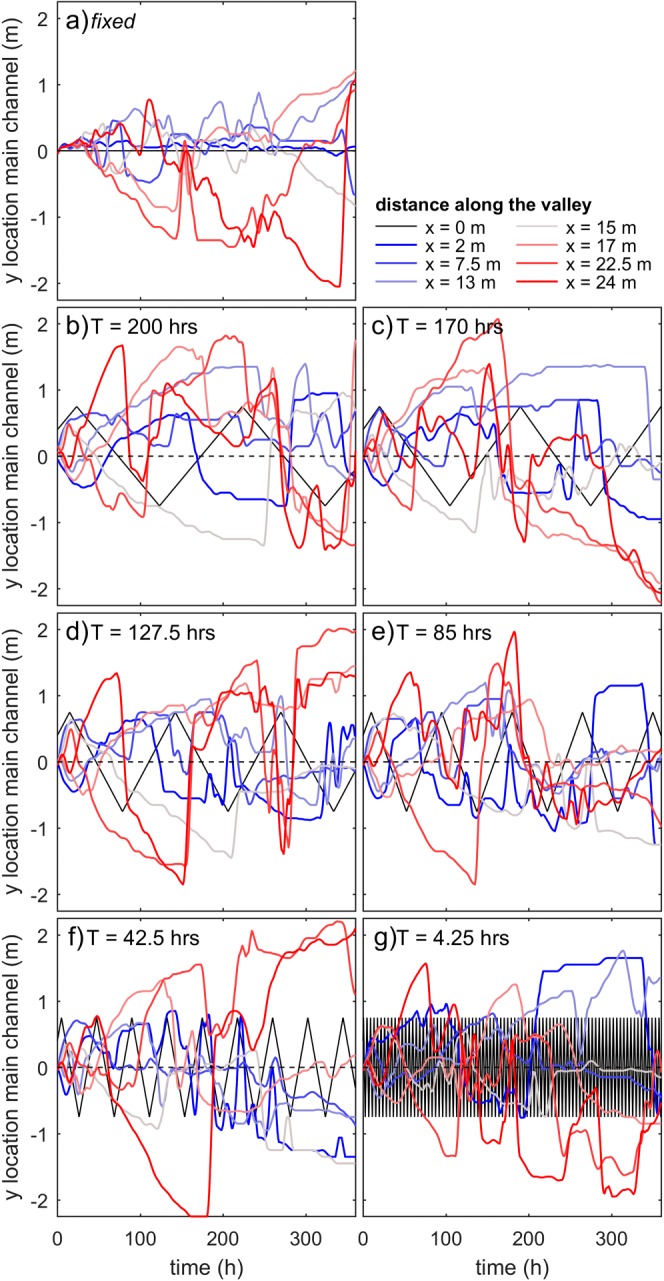
Lateral position of the main channel at several locations along the computational domain for different inlet perturbation periods for comparison with the regularly moving inlet position [Colour figure can be viewed at wileyonlinelibrary.com]

The inflow perturbation period strongly influenced the position and meander period of the first bend (*x*=2 m in Figure 7). A relatively slow inflow perturbation caused cutoff of the forced alternate bar whenever the inflow passed the valley axis, which frequently initiated a cascade of cutoffs in the downstream direction. Such cutoff cascades were also observed in the time series of sinuosity, which gradually increased as bends expanded and abruptly dropped upon cutoff. However, for a relatively fast inflow perturbation, the forced alternate bar developed into a forced mid‐channel bar. This bar pattern decayed in the downstream direction until a dynamically meandering single‐thread channel was attained. Over time, the mid‐channel bar at *x*=2 m widened and increased in height until one of the two channels became the dominant one, irrespective of the location of the inlet (e.g.  *t*>275 h in Figure 7). This resulted in a laterally steady inflow downstream of which new meanders were initiated. The amplitude of the first bend net increased and eventually exceeded the amplitude of the inflow perturbation.

**Figure 8:**
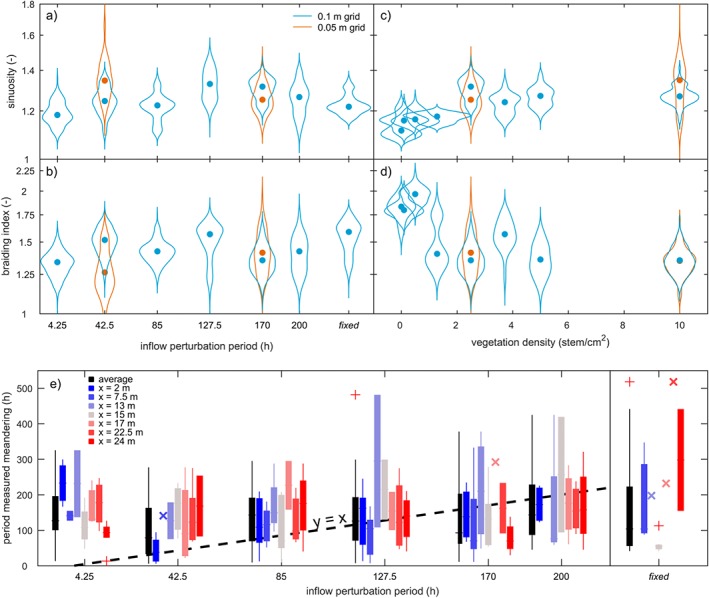
Near‐independence of the internal river dynamics from the inflow perturbation rate. Violin plots show spatially averaged sinuosity and braiding index as function of (a,b) the inflow perturbation period and (c,d) vegetation density, sampled from t=100 h until the end of each model run. (e) No discernible correlation between the period of measured meandering and the period of inflow perturbation. Black bars show the average of seven locations along the valley, of which colours correspond to locations in Figure 7. The ‘×’ gives the meander period when only one could be measured, given model duration [Colour figure can be viewed at wileyonlinelibrary.com]

The bends further downstream migrated uncoupled from the upstream boundary after spin‐up of the model. This was demonstrated firstly by the initiation and cutoff of meanders being unrelated to the periodicity of the inflow perturbation (Figure 7). Secondly, the braiding index and sinuosity were, on average, similar between scenarios (Figure 6). Thirdly, the measured meander period showed no discernible relation with the inflow perturbation period (Figure 8). As a result, large point bars could develop (Figures 2 and 3). Furthermore, the results suggest that fewer cutoffs occurred in scenarios with perturbation periods approximating the typical meander period, but this trend is not clearly significant.

Nonetheless, the dynamic inflow perturbation was necessary to attain and maintain dynamic meandering in the upstream part of the computational grid. This became apparent from scenario N9, in which a static inflow was used. At first, the channel of scenario N9 widened by lateral erosion of both banks. Since the inlet had a fixed width and therefore could not widen, an erratic flow perturbation was generated due to the sudden change in the width‐to‐depth ratio. However, as these perturbations were fairly small, it took approximately 15 m for them to grow and steer new perturbations that were the onset of small meanders (Figure 7a). Also, a static inflow caused the development of levees bordering the main channel near the upstream boundary (Figure 4a). This development stood in stark contrast to the scenarios that included a dynamic inflow perturbation, which developed large meanders close to the upstream boundary.

**Figure 9:**
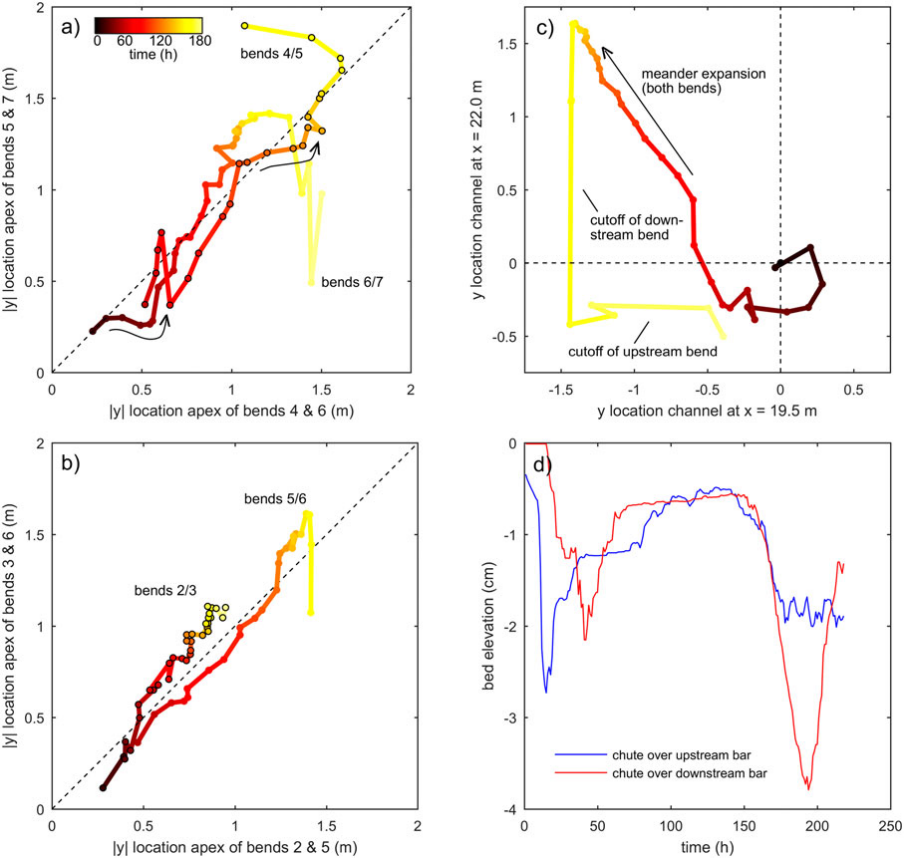
(a) Lateral expansion of two bends in scenario N10 (T=200 h) causes hysteretic expansion of the downstream bend, resulting in concave curve sections, indicated by arrows. Here, bends are counted from x=10 m in the downstream direction. (b) Fairly straight lines of other bends in the same scenario indicate a lack of hysteretic expansion that would suggest a large convective instability. (c) Apparent upstream propagation of perturbation in N10, where cutoff of a downstream bend precedes cutoff of its upstream‐neighbouring bend. (d) Chute channel over upstream point bar (blue; near x=19.5 m) starts forming before the chute channel over the downstream point bar (red; near x=22.0 m). Red becomes the dominant channel, whereas blue remains secondary and is not deeply incised (see text for explanation) [Colour figure can be viewed at wileyonlinelibrary.com]

Perturbations in the modelled domain were found to propagate in the downstream direction. For example, we found that the expansion of a given bend often resulted in the hysteretic expansion of the next bend (Figures 9a and 9b). However, this trend was not clearly observed for all bends. The predominantly downstream effect and strong tendency to form meanders was particularly evident following a cutoff cascade. A large point bar (*x*=9 m at *t*=185 h in Figure 2) developed amidst a weakly braided network, which appeared to filter out the upstream perturbations, but itself caused a steady perturbation that led to meander bend formation in the downstream direction. Oddly, the filtered main channel path sometimes suggested that the cutoff of a bend resulted in the cutoff of its upstream neighbour (Figures 9c and 9d). This would imply that perturbations in the modelled cutoff‐dominated rivers could also propagate in the upstream direction. Upon further inspection, however, the morphological evolution showed that the chute on the upstream point bar was already forming before the chute on the downstream bar. The downstream bar was simply cut off effectively a few modelled years before its upstream neighbour, suggesting that the perceived upstream propagation was coincidental, rather than causally connected.

### Effect of grid resolution

The meander‐scale characteristics were independent of the grid resolution (Figure 8), but the finer grid better resolved features well known from the field. The finer grid enabled deeper incision, which contributed to lower width‐to‐depth ratios. Large tight bends exceeding *π* radials formed more frequently on a finer grid and were predominantly found in the downstream half of the domain. Additionally, point bars on the fine grid showed scrolls and swales and corresponding fine‐grained vegetation age patterns (Figures 2, 3 and 4c), whereas these features were more obscure on a coarse grid.

Grid resolution also affected the distance over which the upstream perturbation effects dampened out. The second fastest perturbation scenario (N13F) showed dampening out over a shorter distance for a fine grid resolution than for a coarse resolution (N13); in particular, there were fewer mid‐channel bars in the upstream reach, resulting in a distinctly lower braiding index and higher sinuosity for the finer grid scenario (Figure 8). Interestingly, the largest sinuosity (1.7) was recorded in scenario N13F, for which the inflow perturbation was over an order of magnitude faster than the typical meander period (Figure 6).

## Discussion

Our model results demonstrate that both bar–floodplain conversion and a sustained dynamic inflow perturbation are needed to reach dynamic meandering in a domain of limited length. Bar–floodplain conversion causes a river pattern transition from braided to meandering, and the dynamic inflow perturbation leads to repeated meander initiation and cutoff close to the upstream boundary, unlike a static inflow. While this is in general agreement with previous findings (Lanzoni and Seminara, 2006; Van Dijk *et al.* 2012; Schuurman *et al.* 2016), the present results for the first time demonstrate that these are necessary and sufficient conditions for sustained meandering: (1) for a large number of bends, unlike previous experiments (e.g. Van Dijk *et al.*, 2013, 2012); (2) for an initially straight channel, unlike previous modelling (e.g. ; Van Oorschot *et al.* 2016); and (3) for entirely self‐formed floodplain, unlike one‐dimensional meander simulators (Lanzoni and Seminara, 2006; Weiss, 2016) and two‐dimensional modelling (Schuurman *et al.* 2016) in which floodplain was taken out of the domain.

The intricate channel belt topography of the simulated rivers is similar in many aspects to that of natural rivers, including features such as compound point bars, chutes, scroll‐like vegetation age patterns and oxbow lakes (Figures 2, 3, 4). These features appear better defined on a finer grid but are generally present at a lower grid resolution as well. The resulting stacked lateral accretion packages and the better preservation of meanders at the flanks of the valley are generally consistent with earlier modelling and experiments (Van de Lageweg *et al.* 2016).

Bar–floodplain conversion was crucial to redirecting flow off the point bars into a single‐thread channel with a low width‐to‐depth ratio (Tal and Paola, 2010; Van Dijk *et al.* 2012; Kleinhans *et al.* 2018). This resulted in bars forced by channel curvature that extended laterally as a consequence of localized outer bend erosion and inner bend accretion (Van de Lageweg *et al.* 2014). In contrast, in less sinuous channels with sparse vegetation, both the gradual erosion of the outer banks and downstream migration of bars suggested that bars migrated more freely. This behaviour implies that effective floodplain formation causes a meandering river pattern. This finding with vegetation as bar–floodplain conversion agent bears resemblance to that of systems with floodplain formation through the sedimentation of fines (Van Dijk *et al.* 2012; Matsubara *et al.* 2015) and the combination of fines and vegetation (Braudrick *et al.* 2009; Constantine *et al.* 2010; Kleinhans *et al.* 2018).

Previous research demonstrated that the river pattern is well predicted by combination of a form of stream power and grain size (Leopold and Wolman, 1957; Furbish, 1988; Kleinhans and Van den Berg, 2011). However, we show that the river pattern is significantly influenced by the degree of bar–floodplain conversion when potential stream power and grain size are invariable (Figure 5). In nature, the stream power of a river sets the boundary conditions for vegetation in a biomorphological feedback loop, such as maximum flow velocity, flooding and desiccation time and water depth. Therefore, the flow regime of a river influences which vegetation species can flourish on the bars, banks and floodplains, which results in a measure of bar–floodplain conversion that is arguably closely related to stream power, as well as grain size. Variation in current relations of stream power, grain size and river pattern (Kleinhans and Van den Berg, 2011) could then be explained by other factors that affect vegetation species‐specific settling, growth and mortality, such as climate and the availability of organic litter and nutrients (e.g. ; Baker, 1989; Bendix and Hupp, 2000).

**Figure 10:**
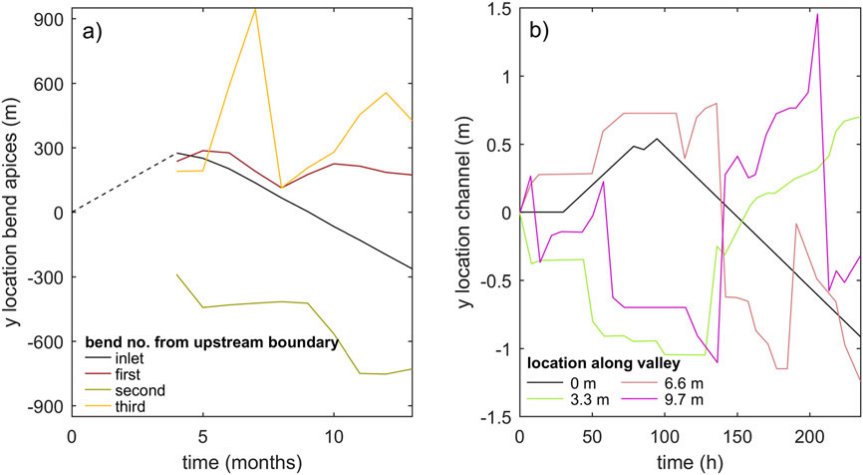
Results from previous studies showing that bends, apart from the first bend, migrate nearly independently from the inflow perturbation (a) in two‐dimensional modelling of high‐amplitude meanders by Schuurman et al. (2016) and (b) in flume experiments by Van Dijk et al. (2012) [Colour figure can be viewed at wileyonlinelibrary.com]

The direct influence of the inflow perturbation period on meandering only extends to the first bend, while all other bends migrate uncoupled from the upstream boundary (Figures 7 and 8). This confirms previous research (Figure 10; Van Dijk *et al.*, 2012; Schuurman *et al.*, 2016) and is attributed to perturbations migrating in the downstream direction and dampening out within a few channel widths (Zolezzi and Seminara, 2001). New bar and bend instabilities are generated at every bend and especially large bends are able to filter out perturbations from upstream (Figure 2) and create a steady perturbation that leads to the onset of meandering in the downstream direction. Consequently, the initiation, migration and cutoff of all bends, apart from the first, are predominantly determined by the inherent biomorphological feedback loops. Also, the amplitude of the inflow perturbation is much smaller than the typical meander amplitude, which corroborates that the meandering dynamics are emergent behaviour rather than forced by the upstream boundary. Nonetheless, it seems that a perturbation period that approximates the typical meander period results in fewer cutoffs, which agrees with recent theory and modelling (Weiss, 2016). Furthermore, applying a periodically rotating rather than a transversely migrating perturbation will likely prevent the first bend and bar from being forced by the inflow perturbation (Weiss, 2016), perhaps reducing the number of cutoff cascades initiated by the inflow perturbation.

Inclusion of both finer sediments that could fill abandoned bends and flow separation at abandoned meanders could have enhanced the persistent dynamic meandering, especially in the models with the fastest inflow perturbations. Firstly, the deposition of fines reduces the accommodation space on point bars by filling in chutes and oxbow lakes, contributing to thick cohesive deposits, and by draping the upper portions of point bars, which decreases bar erodibility. The extent to which such deposition may occur depends on the various plant species at hand, which differ between rivers (Simon and Collinson, 2002; Nicholas, 2013). Consequently, overbank flow is reduced, which could otherwise lead to chute cutoff, as shown in mixed‐load meandering rivers (Braudrick *et al.* 2009; Zinger *et al.* 2013; Kleinhans *et al.* 2018). Secondly, flow separation at abandoned meanders favours the development of a plug bar that reduces the tendency for chute cutoff (Constantine *et al.* 2010; Zinger *et al.* 2013). However, the modelling of flow separation requires much finer grids than is presently feasible; the finest grids required four months of single processor time on a state‐of‐the‐art server PC.

Our findings show that morphology in state‐of‐the‐art physics‐based models requires continuous perturbation to maintain dynamics over the full length of the domain of limited length, which is in agreement with theory and experiments. This insight suggests the need for re‐evaluation of previous experimental, and numerical studies that were terminated before the decay of dynamics, triggered by initial disequilibrium and a static perturbation, could be observed (Friedkin, 1945; Braudrick *et al.* 2009). For meandering, our work shows that the perturbation period has a negligible effect on the river pattern and dynamics, but the dynamic perturbation must be sustained for the persistence of dynamic meandering over the full length of a domain of limited length. An appropriate period of the perturbation is of the order of magnitude of the natural dynamics – i.e., in our case, the meander period.

The present findings also shed new light on the wide scatter observed in relations between meander bend migration and the bend radius normalized by the channel width *R*/*B* (e.g., Hickin and Nanson, 1975; Furbish, 1988; Crosato, 2009). The model results show that the growth and migration of bends resulted in tightening of their downstream neighbouring bends, which in turn affected the migration rate of the latter. Generally, such sharpened bends in our model migrated quickly. However, when bend tightening caused significant overbank flow, the discharge of the main channel decreased, which translated into a smaller strength to erode the outer bank (Van Dijk *et al.*, 2014, 2012) and therefore in slower bend migration. Occasionally, this even caused a cascade of chute cutoffs as was also observed in the River Allier (Kleinhans and Van den Berg, 2011). The downstream propagating effects of bend tightening may explain part of the scatter observed in the aforementioned relations. Backwater and back‐sedimentation effects could theoretically result in upstream propagation of perturbations (Hoyal and Sheets, 2009; Kleinhans *et al.* 2013), but such effects were not observed other than in coincidental cases (Figure 9).

In a real‐world context, external dynamic perturbations are generated by natural processes and repeated human intervention, while internal dynamic perturbations are caused by lateral channel motion at any point along an actively meandering river, contributing to the perpetuation of meandering. Examples of natural dynamic perturbations include an upstream braided river, mass movement from hillslopes, and tributaries shedding water and sediment at different times (Van Dijk *et al.* 2012). Human intervention in rivers introduces new local perturbations that lead to meanders re‐forming over some downstream distance, probably similar to the distance between the first meander and the upstream boundary in our modelling. While many such measures are static (e.g. groynes, dikes), in contrast to ‘soft’ measures such as channel deepening and straightening, they may be destroyed over time, as has happened in the River Otofuke (Figure 1). Consequently, both soft and hard measures collectively provide spatiotemporally dynamic perturbations in the form of local topographic forcing. Downstream of such forcing, the river returns to its inherent dynamic equilibrium. Nevertheless, downstream meander bends indirectly respond to upstream morphological change, implying that human intervention could steer unwanted meander expansion downstream or initiate a cutoff cascade (Hooke, 2004). This could contribute to unwanted faster flood wave propagation, but understanding of the propagation of instabilities can be used in remeandering practices. For example, erosion and deposition are redirected by periodically dumping sediment, retrieved from downstream sediment traps, on different sides of the upstream channel. Thus, the dumping causes a soft dynamic lateral perturbation, as was also demonstrated theoretically by Weiss (2016).

The effects of a sustained perturbation likely extend to other morphodynamic environments. For example, a similar conclusion has been drawn for rip currents in the coastal nearshore zone (Castelle and Ruessink, 2011). They found that a time‐varying wave direction at the seaward boundary of the nearshore causes a natural variability of rip spacing, migration rate and direction (their figure 3). In contrast, when the wave forcing was constant, a steady state with constant wavelength is reached. The most striking dynamics that emerge with varying forcing are commonly observed in nature, i.e. the merging and splitting of bars leading to a change in the number or rip currents. However, no such patterns were reproduced by the model without continued perturbations enforced at one of the boundaries (Castelle and Ruessink, 2011). These findings strongly suggest that a dynamic perturbation on the boundary conditions is necessary in all physical and numerical models for fluvial, estuarine and coastal morphodynamics.

## Conclusions

Morphodynamic modelling with Nays2D was conducted to determine the effect of a dynamic inflow perturbation on the river pattern and dynamics of a dynamic meandering river. This resulted in the following insights.

A dynamic inflow perturbation and bar–floodplain conversion are necessary and sufficient conditions to model dynamic chute cutoff‐dominated meandering in numerical hydromorphodynamic models and in landscape laboratory experiments with a domain of limited length.

Bar–floodplain conversion causes flow to channelize in a single sinuous channel with moderate sinuosity and therefore leads to the meandering river pattern. The model domain spanned about 10 bends, which was sufficiently long to cause re‐formation of meanders following occasional shifts from meandering to weakly braided in part of the domain due to chute cutoff cascades. The resulting sinuosity, braiding index and meander period are predominantly determined by the biomorphodynamic feedback loops, while the effect of the inflow perturbation period is negligible within an order of magnitude of the timescale of meander migration.

Nevertheless, the initiation and persistence of dynamic meandering require a dynamic inflow perturbation or a sufficiently long domain, for bend and bar instabilities propagate in the downstream direction. Although the inflow perturbation period does not have a significant impact on the dynamic equilibrium river dynamics, a perturbation period of the order of magnitude of the main morphological timescale, here meander period, appears to be most appropriate. The implication is that all morphological models and landscape experiments for rivers, estuaries and coasts require perturbations on a boundary in order to model natural morphodynamics.

## Supporting information



Supporting info item
